# Concentric strength training at optimal or short muscle length improves strength equally but does not reduce fatigability of hamstring muscles

**DOI:** 10.14814/phy2.14196

**Published:** 2019-08-18

**Authors:** Katja K. Pedersen, Martin K. Madsen, Lars G. Hvid, Kristian Overgaard

**Affiliations:** ^1^ Department of Public Health, Section of Sport Science Aarhus University Aarhus Denmark

**Keywords:** Fatigue, low frequency fatigue, muscle length specific training

## Abstract

The purpose of this study was to compare the effect of a 6‐week period of knee flexion strength training at either optimal or short muscle length, on length‐specific muscle strength and fatigue. Twelve healthy volunteers performed dynamic (isokinetic concentric) training with one leg at short and the contralateral leg at optimal muscle length for 6 weeks. Knee flexor muscle strength was assessed before and after training, comprising maximal voluntary isometric and dynamic contractions at short, intermediate and near optimal muscle length and electrically evoked, contractions at near optimal length only. Fatigability was tested by performing 60 maximal concentric contractions at either short or optimal muscle length. Isometric torque at all muscle lengths improved equally by training at short and optimal muscle length, for example, tested at short 18 (17) versus 21 (17) % (CI) and at optimal 14 (8) versus 17 (16) % muscle length, respectively. Likewise, equal improvements were observed for dynamic contractions in both groups. Prior to training, fatigue induced at optimal muscle length tended to be more pronounced than at short muscle length (fatigue‐indexes −41 (6) vs. −34 (7) %, respectively, *P* = 0.05). However, training at either length did not reduce fatigability. Training with maximal concentric contractions at either short or optimal muscle length for 6 weeks improved isometric and dynamic muscle strength in the entire range of motion without inducing any discernible length‐specific adaptations. However, strength training at restricted muscle length did not reduce relative fatigue when induced at either short or optimal muscle length.

## Introduction

Muscles are activated at a variety of lengths depending on the specific sports or activities and the muscle in question. During training for such activities, it is usually recommended that movements are carried out at muscle lengths or joint range of motion similar to those occurring naturally in the activity. However, during rehabilitation after injuries to joints or muscles, strength training at a limited range of motion may be used (Osternig, 1986). Therefore, it is of interest to assess whether strength training at a limited muscle length range (or range of motion of the joint) may affect strength and fatigability of the muscle when operating at other muscle lengths.

The majority of studies examining effects of muscle length restricted strength training have observed training‐induced increases in torque to be most pronounced at the length at which muscles were trained (Lindh, 1979; Thepaut‐Mathieu et al. 1988; Graves et al. 1989; Kitai and Sale, 1989; Weir et al. 1994; Kubo et al. 2006; Noorkoiv et al. 2014, 2015). However, such length specificity of strength training was not found in all studies (Rasch and Pierson, 1964; Knapik et al. 1983). The operating length of a muscle may also affect fatigability, as several studies have observed a more pronounced decrease in torque production (Fitch and McComas, 1985; McKenzie and Gandevia, 1987; Lee et al. 1999; Weir et al. 2000; Kooistra et al. 2005; Lee et al. 2007) and increase in low‐frequency fatigue (Jones et al. 1989; Lee et al. 2007) when muscles worked at long or optimal length compared with a shorter length. Most of the abovementioned studies have been conducted using isometric contractions, whereas most in vivo muscle contractions are dynamic. Despite this, only few studies have examined training response to concentric strength training at restricted muscle lengths (Graves et al. 1989; Folland et al. 2005; Valamatos et al. 2018), and these studies have not examined the effects on fatigability at various muscle lengths.

The aims of the present study were, therefore, to examine the effect of a 6‐week period of concentric strength training of the hamstring muscles at either optimal or short muscle length, on length‐specific muscle function and fatigability.

We hypothesized that training at short or optimal muscle length, would increase strength and reduce fatigability most prominently at the trained muscle length.

## Methods

### Ethical approval

The study was approved by the The Ethical Committee of Region Midtjylland (ref. no. 1‐10‐72‐158‐18) and was conducted in accordance with latest version of the Declaration of Helsinki.

### Participants

Fourteen healthy participants (10 males and 4 females) with no lower limb injuries volunteered for the study. None of the participants had performed regular strength training of their lower limbs within the last 6 months, but they were otherwise regularly physically active. Two participants were excluded from the study during pre‐training tests due to non‐completion of test elements, leaving 12 participants (9 males, 25.2 ± 3.8 (SD) years, 183.8 ± 5.9 cm, 76.5 ± 9.4 kg, 18.6 ± 5.5% body fat, and 3 females 25.4 ± 4.0 years, 170. 2 ± 2.1 cm, 70.9 ± 10.4 kg, 31.5 ± 5% body fat). All participants gave a written consent after being informed of the purpose, procedures, and risks of the study.

### Study design

**Figure 1:**
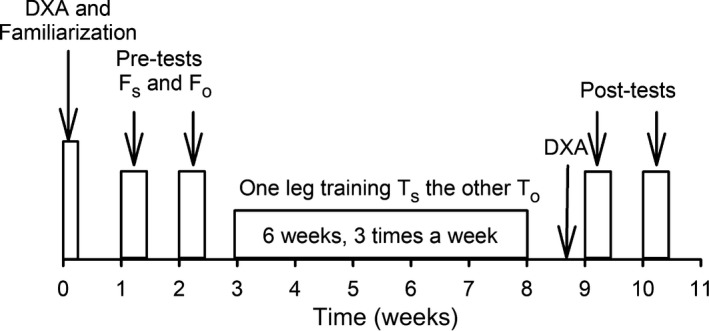
Study design. Participants initially performed a familiarization‐test, followed by the first pre‐test at which both legs were fatigued at either short (*F*_s_ 90–120°) or optimal (*F*_o_ 0–30°) muscle length (randomly assigned). After at least 1 week, the second pre‐test was performed with legs being fatigued using the previously un‐tested muscle length. Hereafter, 6 weeks of training was performed (3 sessions/week, 3 sets of 10 maximal dynamic contractions, 1‐min rest between sets). During training, one leg trained at short (*T*_s_) and one at optimal (*T*_o_) muscle length (randomly assigned). Three days following the last training session, participants performed the first of the two post‐tests followed by the second post‐test one week later in the same order as the pre‐tests. Participants were DXA‐scanned before the first test prior to training and before the first test post‐training.

**Figure 2:**
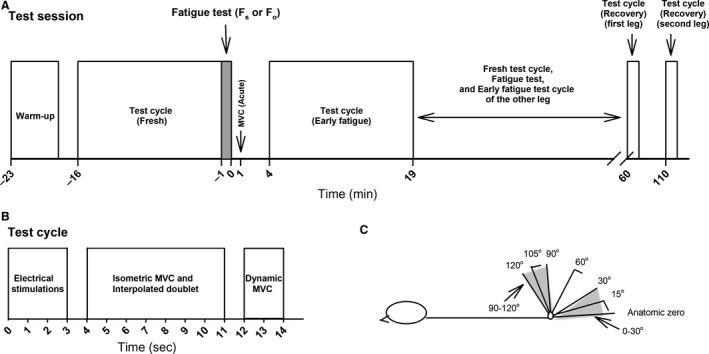
A test session. (A) Test sessions started with warm‐up followed by a test cycle (Fresh) (B), a fatigue test and an MVC within 1 min after fatigue test (Acute). Further test cycles were performed 4 min (Early fatigue) and approximately 1 h following the fatigue‐test (Recovery). Between the Early fatigue test cycle of the first leg and the Recovery test cycle, the second leg was warmed up with heel kicks, and a Fresh test cycle, the fatigue‐test, an Acute MVC and an Early fatigue test cycle were performed. (B) Each test cycle consisted of electrical stimulations, isometric voluntary contractions, and three dynamic contractions in full range of motion. (C) Illustrates the position of the participant on the dynamometer, the test angles, and restricted range of motion for short (90–120°) and optimal (0–30°) length of the muscle.

As shown in Figure 1, participants initially reported to the laboratory for a familiarization session, followed by two testing sessions separated by one week. Participants were randomly assigned to a test order starting with fatigue test of both hamstring muscles at either short or optimal length (Fig. 1), followed by the other length in the second pre‐test performed on a separate day. This order of testing remained the same for each participant during the two post‐tests performed after a 6‐week training period. During the training period, participants trained one leg at short and the other leg at optimal length. Training was performed 3 days/week separated by at least one day of rest. Training bouts consisted of 3 sets of 10 maximal concentric contractions at an angular velocity of 20°/sec with 1.5 sec rest between repetitions, and 1‐min rest between sets. The angular excursions of the knee joint during contractions were 90–120° at short muscle length (*T*_s_) and 0–30° at optimal muscle length (*T*_o_) (Fig. 2).

### Anthropometry

Within one week of the first pre‐test session and 3–7 days after the last training session (yet before the first post‐test session), all participants had their body composition and thigh fat‐free mass assessed by dual‐energy X‐ray absorptiometry (DXA) (Lunar IDXA, GE Healthcare, Madison, WI). All DXA‐scans were carried out after overnight fasting.

### Measurement of torque

All tests and training were performed using an isokinetic dynamometer (Humac Norm, CSMI, Stoughton, MA), and all position and torque data were sampled at 1500 Hz using TeleMyo Direct Transmission System and MyoResearch Software (Noraxon, Scottsdale, Arizona, USA). Participants lay in a prone position with a velcro strap securing the lower back/hips while the leg was fastened to the dynamometer arm with the knee joint axis (the lateral femoral condyle) aligned with the rotation axis of the dynamometer. Anatomical zero (stretched knee, Fig. 2C) is referred to as 0°, with the test angles for isometric maximal voluntary contractions (MVC) being 15°, 60°, and 105°. Pilot studies showed an average isometric optimal angle of approximately 15° which is referred to as “near optimal angle”, whereas 60° is referred to as intermediate angle and 105° as a short angle, respectively. For maximal voluntary isokinetic concentric knee flexion contractions performed at a velocity of 20°/sec, angular excursion from 0 to 30° was referred to as optimal muscle length and 90–120° as short muscle length. All test contractions were separated by 1 min, and standardized verbal encouragement was provided at each contraction. In addition to tests of maximal voluntary isometric/dynamic contractions, a muscle fatigue‐test was carried out, consisting of 60 maximal concentric contractions, each contraction separated by 1.5 sec, at either short (*F*_s_ 90–120°) or optimal (*F*_o_ 0–30°) muscle length.

### Electrical stimulation and EMG measurements

Muscles were electrically stimulated to produce isometric torque at the “near optimal angle” of 15°. After shaving and cleansing the skin with alcohol swabs, two electrodes (ValuTrode Neurostimulation Electrodes VL4595, 2" × 4", Axelgaard Manufacturing Co., Ltd, Denmark) were placed across the muscle belly of biceps femoris interspaced with a distance of 6–9 cm, depending on muscle size of the participant. Surface electromyography (EMG) was measured by placing two electrodes (Ambu Blue Sensor N, AMBU, Ballerup, Denmark) lengthwise medial on the longitudinal line of biceps femoris with an interelectrode (center‐to‐center) distance of 20 mm. This was according to SENIAM recommendations (Hermens et al. 2000). Electrode positions and anatomical landmarks were marked on to a transparent sheet during the familiarization session, and individual sheets were used to replicate the electrode position during subsequent tests. An individual level of current for electrical stimulation was determined during the familiarization session, by increasing current of stimulation using doublets of 100 Hz until a torque plateau was reached, without exceeding the acceptable pain tolerance level for the participant. This current ranged from 300 to 425 mA, and was used for doublet stimulation, while in train stimulations half of this individually determined current was used in order to avoid excessive pain from stimulation. The individually determined current for each participant was maintained at the same level for all test sessions, and furthermore, to become accustomed to the electrical stimulation at a test, current was gradually increased initially in each session until the pre‐determined level was reached.

To assess low‐frequency fatigue, 15 and 50 Hz stimulation trains were applied (duration 0.4 sec), which produced a partly fused and a completely fused contraction, respectively. In addition to these measurements, assessment of the activation level was performed using an MVC with an interpolated doublet, but due to low quality of these data, they are not presented.

### Test sessions

The participants were instructed to refrain from strenuous exercises 24 h prior to reporting to the laboratory for testing. The overall design of each test session is depicted in Figure 2A. At arrival, participants performed a 5 min warm‐up, cycling on a stationary ergometer bike (Monark Ergomedic 828E, Monark, Sweden). As a further warm‐up procedure before dynamometer testing, the participants performed 10 forceful “heel to backside” kicks, with the first leg to be tested. Additionally, after positioning in the dynamometer, three isometric warm‐up trials at 15° were performed at intensities of 50, 75, and 100% of MVC. Tests were organized into cycles, and each test cycle consisted of electrical stimulations followed by MVC at 15°, 60°, and 105° (Fig. 2B). Lastly, participants performed three successive dynamic contractions in the entire range of motion (from 0° to maximal flexion). The first test cycle (Fresh) was applied 2 min after a gradual current increase to the predetermined level, followed by the dynamic fatigue‐test at either short or at optimal muscle length. Within 1 min after finishing this fatigue‐test, an acute MVC at 15° was performed (Acute). Three min rest was allowed before starting the second test cycle, with electrical stimulation and MVC’s, see Figure 2B (Early fatigue). Between the Early fatigue test cycle and the third test cycle 1‐h postfatigue (Recovery), the other leg performed the same sequence of tests and fatiguing contractions. Between the two Recovery test cycles, participants were allowed to sit up or walk around lightly. For the Fresh part of the test cycles, the participant performed two trials for MVC at 15°, 60°, and 105°. If difference between trials exceeded 10%, a third trial was performed. If the difference then was more than 10% between the two best trials, only the best trial was used.

### Data analysis

The data were analyzed using commercial software (Math Works, Matlab R2017b, MA). Data were filtered using a second‐order Butterworth filter with a cut‐off frequency of 5 Hz, except for records of electrical stimulation, which were analyzed unfiltered. Recordings showing measurement artefacts, which precluded proper measurements, were discarded. Voluntary and electrically evoked peak torque (*Nm*) was determined for each isometric contraction. Peak torque and work (*J*) were calculated for dynamic contractions by averaging the peak torque and work produced in valid recordings over the entire angular range of motion and for the two first contractions in the angle‐restricted fatigue‐tests. A fatigue‐index during dynamic contractions was determined from the percentage decrease in work produced between the first two and last two contractions of the fatiguing protocol. The raw EMG signal was analyzed with a Root Mean Square filter (EMGrms), with a window length of 500 msec. EMGrms was measured for isometric MVC of 15° and 105° over the 500 msec leading up to the detected peak torque, and for dynamic (concentric) contractions at restricted muscle lengths over a 500 msec time window around the optimal muscle length (corresponding to the movement from 10° to 20° knee angle) and the short muscle length (corresponding to the movement from 100° to 110° knee angle), respectively.

All data are expressed as means ± SD (tables) or 95% confidence intervals (CI used in figures, text and where indicated in tables). Statistical analyses were carried out using a linear mixed model (STATA10.1, StataCorp, USA). All data were shown to be normally distributed. Measurements of isometric torque at 15°, 60°, 105°, dynamic peak torque, dynamic work, low‐frequency fatigue, and EMGrms were analyzed with Group (*T*_o_, *T*_s_), Time1 (Pre, Post‐training), Time2 (Fresh, Acute, Early fatigue, Recovery), and Fatigue (*F*_s_, *F*_o_) set as fixed effects. The main outcome was Group*Time1 interactions (within‐group from Pre to Post, separately for *T*_s_ and *T*_o_, and between‐group at Pre), Fatigue*Time1 interaction (at Pre and Post, respectively, and at *F*_s_ and *F*_o_), as well as Fatigue*Time2 interaction (at Pre and Post, for *F*_s_ and *F*_o_). We observed an increase in MVC torque from the first to the second pre‐test, likely representing a learning effect. To avoid influence from this, pre‐training values obtained in the second pre‐test were compared to the first post‐test for testing significance of training effects. Statistical significance was accepted at *P* < 0.05.

## Results

Both legs completed 17.5 ± 0.5 of 18 training sessions, with the same number of contractions performed in each leg. However, the leg training at short muscle length performed less work compared with the leg training at optimal length (69 (20) vs. 120 (35) *J* (SD), respectively, *P* < 0.001).

**Figure 3:**
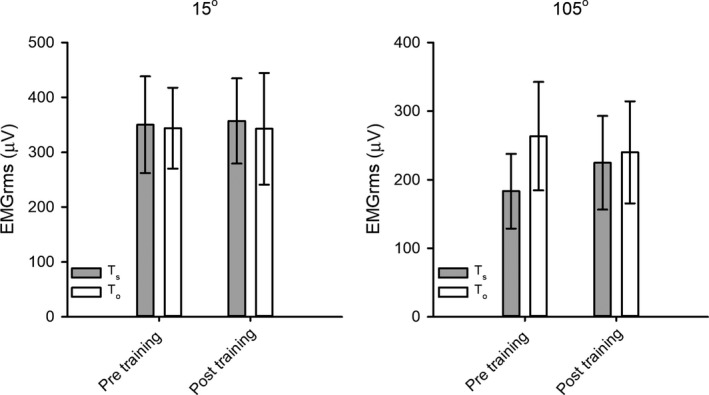
EMGrms during maximal isometric voluntary contractions at 15° and 105°. Data are presented as means with error bars denoting ±CI. *n* ranged from 11 to 12.

Concentric hamstring training for 6 weeks at either short (*T*_s_) or optimal (*T*_o_) muscle length showed a significant within‐group improvements in isometric torque at 15°, 60°, and 105°, but with no between‐group difference in the magnitude of improvement at any of the measured angles (Table 1). Furthermore, electrically evoked torque at 15 and 50 Hz increased with training after *T*_o_, while a similar tendency toward an increase was observed at 50 Hz when trained at *T*_s_. Thus, no between‐group difference was seen in the change in electrically evoked torque between *T*_o_ and *T*_s_ (Table 1). Furthermore, as shown in Figure 3, no within‐group or between‐group differences following training were observed for EMGrms at isometric MVC 15° and 105°. Comparable finding were observed for EMGrms for dynamic contractions, that is, with no within‐group or between‐group differences following training (data not shown). As an indicator of central (nervous system) adaptations, we furthermore did not observe within‐group training effects in torque elicited by 50 Hz stimulation expressed as percentage of MVC (50 Hz % MVC), at either training length. As an indicator of peripheral (fat‐free mass) adaptations, a DXA‐scan of each thigh (combined fat‐free mass of anterior and posterior muscle groups) did not show any significant within‐group training‐induced differences in fat free mass for either *T*_s_ or *T*_o_.

**Table 1:** Isometric training response to concentric training at short (*T*_s_ 90–120°) or optimal (*T*_o_ 0–30°) muscle length for 6 weeks.

	T_S_				T_O_				Group comparison
	Pre	Post	Change % (CI)	*P*	Pre	Post	Change % (CI)	*P*	*P*
MVC 15° (*Nm*)	115 (25)	131 (28)	14 (8)	0.006	113 (27)	132 (31	17 (16)	<0.001	0.7
MVC 60° (*Nm*)	90 (19)	104 (24)	18 (16)	0.03	85 (17)	106 (29)	25 (17)	0.006	0.6
MVC 105° (*Nm*)	53 (12)	61 (15)	18 (17)	0.03	50 (9)	61 (16)	21 (17)	0.002	0.8
15 Hz (*Nm*)	21 (10)	26 (9)	44 (52)	0.1	23 (8)	28 (8)	32 (31)	0.04	0.7
50 Hz (*Nm*)	29 (12)	35 (10)	30 (30)	0.06	29 (11)	36 (11)	33 (31)	0.04	0.9
50 Hz % MVC	25 (8)	27 (8)	12 (19)	0.4	26 (8)	27 (6)	15 (29)	0.6	0.8
DXA thigh FFM (*kg*)	6.9 (1.3)	6.9 (1.3)	0.3 (3.1)	0.7	6.8 (1.3)	6.9 (1.4)	1.5 (3.9)	0.2	0.5

Pre‐ and post‐training values of maximal voluntary contractions (MVC) measured at 15°, 60° and 105°, and electrically evoked torque at 50 and 15 Hz at an angle of 15°. 50 Hz stimulation expressed as % of MVC at 15° (50 Hz % MVC). DXA‐scan thigh fat free mass (FFM) is a combined result of both the front and back of the thigh. Data are presented as mean (SD) unless otherwise stated. *n* = 12.

As shown in Table 2, significant within‐group changes with training were observed for dynamic function, expressed as peak torque and work. As with isometric torque mentioned above, the improvements were similar for both training groups (i.e., with no between‐group difference), and were present at both short and optimal muscle lengths, indicating no length specificity of training.

**Table 2:** Dynamic training response to concentric training at short (*T*_s_ 90–120°) or optimal (*T*_o_ 0–30°) muscle length for 6 weeks.

	*T*_S_				*T*_O_				Group comparison
	Pre	Post	Change % (CI)	*P*	Pre	Post	Change % (CI)	*P*	*P*
Peak torque_total‐ROM_ (*Nm*)	89 (24)	99 (28)	11 (8)	0.008	88 (21)	99 (25)	13 (9)	0.007	0.7
Work_Total‐ROM_ (*J*)	352 (77)	392 (103)	11 (8)	0.01	343 (89)	396 (102)	16 (10)	0.006	0.4
Peak torque_90–120_ (*Nm*)	44 (11)	58 (17)	33 (13)	<0.0001	41 (11)	57 (17)	38 (10)	<0.0001	0.3
Work_90–120_ (*J*)	50 (13)	69 (20)	41 (22)	0.0009	44 (10)	68 (18)	54 (15)	<0.0001	0.2
Total work ‐ fatigue session_90–120_ (*J*)	2086 (460)	2985 (905)	45 (25)	0.002	1932 (448)	3027 (824)	57 (18)	<0.0001	0.2
Peak torque_0–30_ (*Nm*)	79 (19)	91 (26)	15 (14)	0.03	82 (24)	91 (24)	13 (10)	0.03	0.8
Work_0–30_ (*J*)	109 (26)	124 (37)	15 (17)	0.0497	108 (33)	125 (35)	17 (9)	0.002	0.7
Total work ‐ fatigue session_0–30_ (*J*)	4270 (964)	5093 (1350)	20 (14)	0.004	4369 (1068)	5128 (1426)	18 (13)	0.006	0.8

Peak torque and work for dynamic contractions in total range of motion (Peak Torque_Total‐ROM_ and Work_Total‐ROM_). Peak torque and work for the first two contractions in angle‐specific range of motion from 90 to 120° (Peak Torque_90–120°_ and Work_90–120_) and from 0 to 30° (Peak Torque_0–30_ and Work_0–30_), and total work produced during fatigue‐tests at short length (Total work − fatigue session_90–120_) and optimal length (Total work − fatigue session_0–30_). All data are presented as mean (SD) unless otherwise stated. *n* = 12.

Regardless of training regime, the training period induced a comparable increase in the absolute work performed in maximal contractions at short and optimal muscle length (~15–24 *J* increase – see Table 2). However, as the work produced in contractions at short length was initially much lower than at optimal length (50 (13) vs. 108 (33) *J* (SD)), the relative increase in work was greater when measured at short muscle length (corresponding to +41 ± (22)% (CI)) than at optimal muscle length (corresponding to +17 (9)% increase).

**Figure 4:**
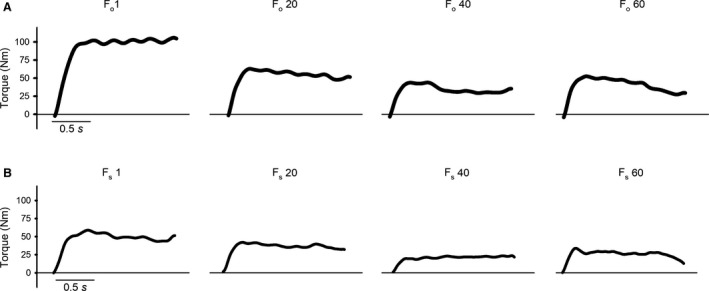
Torque production in dynamic contractions during fatigue. Representative torque traces from the 1st, 20th, 40th, and 60th contraction in the fatigue sessions at optimal muscle length (*F*_o_) (A) and at short muscle length (*F*_s_) (B). The records were obtained from the right leg of one participant, performing the fatigue sessions prior to the training period.

**Figure 5:**
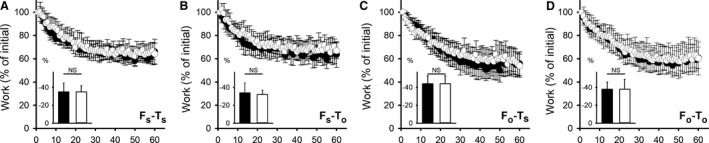
Fatigue development at short and optimal muscle length. Work as percentage of initial work (1st contraction of 60). Closed symbols represent pre‐training, open symbols represent post‐training values. (A) Muscles fatigued at short and trained at short muscle length (*F*_s_‐*T*_s_). (B) Muscles fatigued at short and trained at optimal muscle length (*F*_s_‐*T*_o_). (C) Muscles fatigued at optimal and trained at short muscle length (*F*_o_‐*T*_s_). (D) Muscles fatigued at optimal and trained at optimal muscle length (*F*_o_‐*T*_o_). Inserts represent fatigue‐indexes pre‐ and post‐training. Data are presented as means with error bars denoting ±CI. *n* = 12.

As shown in Figures 4 and 5, 60 maximal contractions induced fatigue at both short (90–120°) and optimal muscle length (0–30°). Fatigue‐indexes for *F*_s_ and *F*_o_ did not change significantly due to strength training at either length, *F*_s_ − T_s_, *P* = 0.98, *F*_s_ − *T*_o_, *P* = 0.7, *F*_o_ − *T*_s_, *P* = 0.97, *F*_o_ − *T*_o_, *P* = 0.7 (Fig. 5). To compare fatigue induced at short and optimal muscle length prior to the training period, data from both legs were pooled regardless of training allocation. These pooled data displayed a tendency to a larger relative fatigability at optimal than at short length (−41 (6) vs. −34 (7) %, respectively, *P* = 0.05), as can be seen when comparing panels A and B with C and D in Figure 5.

**Figure 6:**
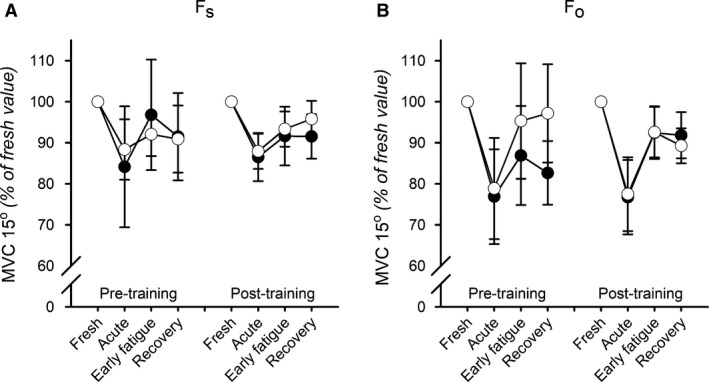
Effects of fatigue elicited at different muscle lengths on isometric torque, pre‐ and post‐training. Isometric torque measured at near optimal angle of 15° and presented as percentage of values before fatigue (Fresh). Measurements were performed pre and post a 6‐week period of training at either short muscle length (closed symbols) or at optimal muscle length (open symbols). Fatigue was induced by 60 maximal concentric contractions at either short (*F*_s_, A) or optimal (*F*_o_, B) muscle length. Data are presented as mean with error bars denoting ±CI. *n* ranged from 8 to 12.

As shown in Figure 6, the fatiguing contractions elicited an immediate decrease in isometric torque measured at optimal length. The fatigue‐induced decrease averaged 12–16% after fatigue at short muscle length compared with 21–23% after fatigue at optimal muscle length, but with no significant differences between *F*_o_ and *F*_s_ in any group. A gradual, but slow recovery was seen over the following minutes, but torque was still suppressed after 1 h of recovery in all conditions (except for pre‐training *F*_s_ − *T*_s_ and *F*_o_ − *T*_o_ at Early fatigue and Recovery, respectively). No significant differences were observed between fatigue patterns pre‐ and post‐training for *T*_s_ and *T*_o_ at neither *F*_s_ nor *F*_o_.

**Figure 7:**
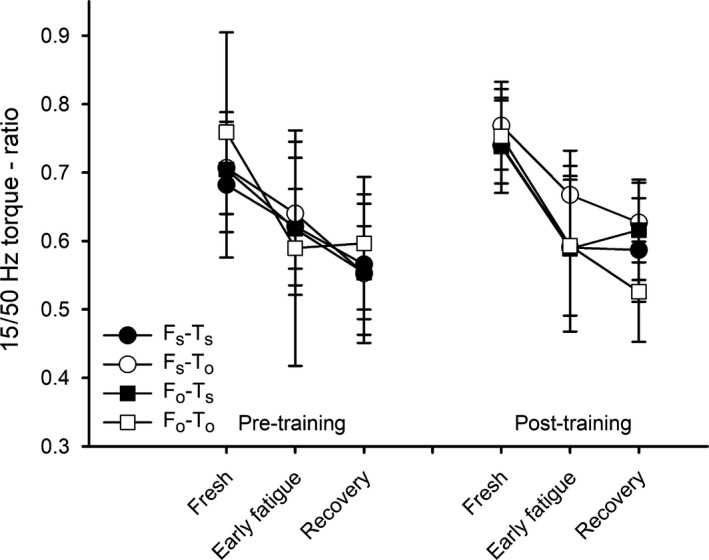
Low‐frequency fatigue following concentric fatiguing contractions. The torque ratios between 15 and 50 Hz are presented for Fresh, Early fatigue, and Recovery after fatigue at either optimal or short muscle length (*F*_o_ and *F*_s_), for legs trained at either optimal or short muscle length (*T*_o_ and *T*_s_) prior to and after 6 weeks of concentric training (Pre‐ and Post‐training). Data are presented as mean with error bars denoting ±CI. *n* ranged from 8 to 12.

We used the 15/50 Hz ratio as an indicator of low‐frequency fatigue and observed that the ratio was reduced both at Early fatigue and at Recovery, after inducing fatigue at either optimal or short length. However, no differences were observed in 15/50 Hz ratio between *F*_o_ and *F*_s_, indicating that the degree of low‐frequency fatigue was equal after fatigue, induced at either short or optimal length (Fig. 7). Training at either length did not influence the low‐frequency fatigue response (Fig. 7).

## Discussion

The main findings of the present study are that strength training with maximal concentric contractions for 6 weeks at short or optimal muscle length (= range of motion) increased torque and work in both isometric and dynamic contractions, but, surprisingly, without any length specificity. Also, length restricted training did not reduce fatigability at either the trained or the non‐trained muscle length. Lastly, development of low‐frequency fatigue occurred after concentric fatiguing contractions to a similar degree at either short or optimal length and was not reduced by training at either muscle length.

### Effects of training on strength at optimal and short muscle length

The finding that training induced strength gains that were not length specific complies with some previous findings suggesting that training at either optimal or long muscle length is capable of inducing significant training effects at all tested muscle lengths (Rasch and Pierson, 1964; Thepaut‐Mathieu et al. 1988; Kubo et al. 2006). However, most studies have observed better training related effects at and nearby the length/angle at which the training was performed (Lindh, 1979; Thepaut‐Mathieu et al. 1988; Kitai and Sale, 1989; Kubo et al. 2006; Noorkoiv et al. 2014, 2015).

A possible explanation for this discrepancy may be that, the present study used concentric isokinetic contractions for training, while most previous studies examining length specificity of strength training used isometric contractions (Rasch and Pierson, 1964; Lindh, 1979; Thepaut‐Mathieu et al. 1988; Kubo et al. 2006; Noorkoiv et al. 2014, 2015). The lack of a length specificity of training may be related to this difference in contraction mode, as different adaptations to isometric than concentric strength training is a possibility (Duchateau and Hainaut, 1984). However, like other studies, the present study did observe a transfer of training effect from slow concentric training to an increase in isometric strength (Knapik et al. 1983; Graves et al. 1989; Weir et al. 1997). Our study shows that such transfer of training effect between concentric and isometric modes may extend to muscle lengths beyond those at which training was performed. This is in accordance with Graves et al. (1989), who performed a study where participants trained concentrically with knee extensions at either 0–60°, 60–120°, or 0–120° and showed, in accordance with the present study, increases in isometric torque at all testing angles, also outside of training ROM (although adaptions were significantly larger at angles within training ROM). We observe in the present study that the non‐length specific training effect applied to torque and work produced in dynamic contractions as well. Since training was performed at two different length ranges in the two legs of each subject, it is possible that a cross‐over effect (Weir et al. 1994, 1997; Carroll et al. 2006) might partially explain the lack of length specificity. A review reported that unilateral training induces increases in strength in the contralateral (untrained) leg corresponding to as much as half of the increase in strength of the trained leg (Carroll et al. 2006). Altogether, they conclude that the contralateral training effect is real, but small (Carroll et al. 2006). However, three previous studies showed length‐specific effects of isometric training despite using a similar design as in the present study (training simultaneously one leg short and the other leg long, for 6 weeks) (Lindh, 1979; Noorkoiv et al. 2014, 2015), furthermore a study using concentric knee extensions in partial and full ROM, also showed length‐specific effects (Valamatos et al. 2018). Even though results of these studies argue against that the cross‐over effect would be large enough to counteract the length‐specific effects, we cannot exclude a possible minor cross‐over effect in our study. Another explanation for our findings could be related to the nature of adaptations induced by the strength training. Strength training has been shown to increase neural activation, especially within the first weeks of training (Thepaut‐Mathieu et al. 1988; Kitai and Sale, 1989; Weir et al. 1994; Del and Cafarelli, 2007). Some studies find evidence that length specific training induces a length specific increase in the activation response (Thepaut‐Mathieu et al. 1988; Noorkoiv et al. 2014), while others do not find the same specificity (Weir et al. 1994; Kubo et al. 2006). But, we did not observe any effect of training on EMGrms at either length when tested by MVC’s, and in addition, we observed training‐induced increases in electrically evoked torque, which were about the same relative magnitude as the increase in MVC. Thus, training induced no differences in 50 Hz expressed as percentage of MVC (50 Hz % MVC), an indicator of central (nervous system) adaptations. This suggests that adaptations in strength may to a large extent have been due to peripheral changes to the muscle, rather than changes in neural activation (Duchateau and Hainaut, 1988). Nevertheless, we cannot exclude the occurrence of neural adaptations as well, since our EMGrms data display a relatively large variation. Furthermore, strength training is known to elicit increases in muscle mass (owing to increases in fiber cross sectional area (Wernbom et al. 2007), thus partly contributing to an increase in muscle strength independently of muscle length. However, we did not observe any changes in DXA‐assessed thigh fat‐free mass following training at either length. This may be explained by the fact that 6 weeks of strength training may be too short a period for muscle mass gains to become evident (Wernbom et al. 2007). Moreover, it should be noted that with this method small changes in hamstring muscle size might go unnoticed, because this measurement represents the entire thigh, of which only a fraction is made up of the trained hamstring muscles.

An important element to emphasise is that the work performed during training at the two different muscle lengths differed. More work was performed during optimal than short length training, despite an equal number of maximal repetitions performed. In light of this, it is interesting that similar non length‐specific strength gains were observed for both training lengths. This indicates that activation of the muscle with intended maximal effort during training is an important element in the stimulus for strength gains, regardless of the work/torque produced in the contractions, a finding which in some aspects is analogous to findings with blood flow restricted training, where significant strength gains can be achieved despite the load lifted is considerably lower than in conventional strength training (Wernbom and Aagaard, 2019).

We noted that the training (at either length) had a similar absolute, but larger relative effect on contractile work capacity when tested dynamically at the short muscle length than at the long length. We speculate that this finding may be explained by the fact that hamstring muscles are rarely activated maximally at very short length. Therefore specifically activating the hamstring muscles concentrically with maximal effort may have induced an additional learning effect at the short length, since it was a contraction modality the participants were unaccustomed to and found difficult to perform.

The finding that training concentrically with maximal contractions at restricted muscle lengths can induce similar strength gains at other muscle lengths beyond the training range, could be of relevance during, for example, rehabilitation of patients or athletes that are restricted in their ROM by an injury to muscles or joints. This type of training might be able to aide in maintaining or improving strength during injury periods, before it is possible to train in the full ROM again. Whether this applies to other muscles than the hamstring muscles and in rehabilitation regimes remains to be examined.

### Fatigue induced concentrically at short and optimal length, and the response to training

We found a tendency towards reduced fatigability at short length compared to optimal muscle length when fatigue was induced concentrically. This corresponds well with previous findings on fatigue induced by isometric contractions (Fitch and McComas, 1985; McKenzie and Gandevia, 1987; Lee et al. 1999; Weir et al. 2000; Kooistra et al. 2005; Desbrosses et al. 2006; Lee et al. 2007). Possibly this difference relates to a smaller number of active cross‐bridges during contractions at short than at optimal muscle length (Aljure and Borrero, 1968; Fitch and McComas, 1985; Ng et al. 1994; Lee et al. 2007). Accordingly, in the present study, work produced during the fatigue‐test elicited at suboptimal muscle length (*F*_s_) was only a little more than half the work produced during optimal muscle length (*F*_o_), which could explain the difference in fatigability. Arguing against this possibility, however, two studies measuring ATP consumption by use of ^31^P‐NMR spectroscopy, found no difference between fatigue induced at optimal and short muscle lengths (Baker et al. 1992; Sacco et al. 1994). Other factors apart from differences in metabolism may contribute to length differences in fatigue. Thus, fatigue at short muscle length has been ascribed to activation failure and fatigue at long length to contractile failure (Weir et al. 1996).

It has previously been shown, that fatigue induced in one leg can influence fatigue induced in the contralateral leg (Amann et al. 2013); however, while the time between the two fatiguing tests in the study by Amann et al. 2013 was only 2 min, the time between fatiguing tests in our study were 35–45 min. Additionally, we did not find any significant difference between the first and the second fatigued leg in neither fatigue‐indexes, initial isometric torque at 15° nor in percent decrease in isometric torque at 15° from Fresh to Acute, at either *F*_s_ or *F*_o_ (data not shown). This indicates that the second tested leg was not affected by the fatigue of the first leg tested.

Training at either *T*_s_ or *T*_o_ led to a significant increase in work produced during the fatigue‐test after the training period at both *F*_s_ and *F*_o_, because both training regimes increased concentric strength. However, in contrast to our hypothesis and a previous strength training study (Gacesa et al. 2013), the fatigue‐indexes for both muscle lengths were not reduced after 6 weeks of training. A possible explanation for this lack of reduction in fatigability could be that the training program consisted of 30 contractions divided in 3 sets pr. training‐day, while the fatigue‐test examined fatigability over 60 consecutive maximal contractions. We therefore examined the relative decrease in work produced during the first 10 and 30 contractions of the fatigue‐test. But also in these analyses, we observed unaltered fatigue‐index post‐training, independent of muscle length during fatigue or during training (data not shown). The present study examined relative decrease in work using 60 maximal concentric contractions, finding no difference pre‐ and post‐training. However, we did observe an increase in total work performed during the fatigue‐test, which is similar to other findings (Gacesa et al. 2013), indicating that muscles worked at a higher absolute load throughout the fatigue‐test. Previous studies have observed less fatigue development following a period of strength training, when using the same absolute load before and after training to fatigue the muscles (Izquierdo et al. 2009), or as an increased time to fatigue with increasing load (Kemi et al. 2011). In contrast, however, two previous studies have shown that using the same relative load of 80–90% of 1RM pre‐ and post‐training, resulted in a more pronounced fatigue following training (Izquierdo et al. 2009; Mayhew et al. 2011). This indicates that the mode of testing may influence the outcome with respect to training effects on fatigability.

### Low‐frequency fatigue

Long‐lasting low‐frequency fatigue was induced by our fatigue protocol at both muscle lengths. However, in contrast to our hypothesis, low‐frequency fatigue was similar following fatigue induced at short and optimal muscle length. Furthermore, no adaptions were found after a training period of 6‐weeks, at either short or optimal muscle length. The lack of difference in low‐frequency fatigue response between short and optimal muscle length is in contrast to the study by Lee et al. 2007, who showed more pronounced low‐frequency fatigue at optimal muscle length than at short muscle length (Lee et al. 2007). However, this discrepancy could be due to experimental design, where Lee et al. 2007 used electrical stimulation to induce fatigue, in an isometric setting, differing from the present study using concentric voluntary activation. This difference between fatigue induced by electrical stimulations and voluntary contractions may also explain why the results of the present study differed from previous observations from our laboratory, where a substantial low‐frequency fatigue was developed in rat soleus muscles when fatigued with shortening contractions to below 50% of optimal length, but not at longer lengths (Overgaard and Nielsen 2004).

Low‐frequency fatigue has been ascribed to failure of the excitation‐contraction coupling, with a decreased release of Ca^2+^ (Westerblad, Duty, and Allen 1993; Westerblad et al. 2000). Although it has been suggested that Ca^2+^ sensitivity is decreased at shortened muscle length (Stephenson and Wendt, 1984), this was contradicted by Balnave and Allen 1996, who implied that Ca^2+^ sensitivity cannot simply be related to variation in muscle length, but may be related to the cross‐bridge attachment and/or force development at particular muscle lengths (Balnave and Allen, 1996). A different result of low‐frequency fatigue might therefore have been obtained if evaluated at short muscle length (105°). However, pilot‐studies showed that muscles had a tendency to cramp when stimulated at short length, which precluded such measurements. Furthermore, it is possible that changes in low‐frequency fatigue could have been induced by a different training regime more focused on fatigue‐resistance, but this remains to be examined.

## Conclusion

In conclusion, the present study shows that concentric training for 6 weeks at either short or optimal muscle length can improve isometric and dynamic torque in a non‐length specific manner, as function improved equally at all tested muscle lengths. However, following training at either length, muscle fatigued to the same relative degree as before training, indicating no length‐specific effects of training on fatigability. Furthermore, low‐frequency fatigue was similar at short and optimal muscle length and was not affected by training at either length. The results indicate that length restricted concentric strength training may be an effective mode of training in situations where the full range of motion cannot be achieved.

## Conflict of Interest

No conflicts of interest, financial or otherwise are declared by the authors.
